# Deep Learning-Based Iodine Contrast Augmentation for Suboptimally Enhanced CT Pulmonary Angiography: Implications for Pulmonary Embolism Diagnosis

**DOI:** 10.3390/diagnostics15182325

**Published:** 2025-09-13

**Authors:** Kyungsoo Bae, Tae Hoon Kim, Kyung Nyeo Jeon

**Affiliations:** 1 Department of Radiology, Institute of Medical Science, School of Medicine, Gyeongsang National University, Jinju 52727, Republic of Korea; ksbae@gnu.ac.kr; 2 Department of Radiology, Gyeongsang National University Changwon Hospital, Changwon 51472, Republic of Korea; 3 Department of Internal Medicine, School of Medicine, Gyeongsang National University, Gyeongsang National University Changwon Hospital, Changwon 51472, Republic of Korea; plm.dr.th.kim@gmail.com

**Keywords:** pulmonary embolism, CT, deep learning, iodine, contrast agent

## Abstract

**Background/Objectives**: This study aimed to assess the impact of a deep learning-based iodine contrast augmentation (DLCA) algorithm on image quality and diagnostic performance for pulmonary embolism (PE) detection in suboptimally enhanced CT pulmonary angiography (CTPA). **Methods**: We retrospectively included 103 suboptimal CTPA cases performed between May 2020 and March 2025. Image quality (attenuation, noise, SNR, and CNR) was compared between original and DLCA-processed images. Diagnostic performance for PE detection was assessed per segment, with and without DLCA processing. **Results**: DLCA increased pulmonary artery opacification by 57.7% and reduced noise by 56.7%, significantly improving SNR (13.2 → 47.5) and CNR (8.7 → 37.2; both *p* < 0.001). Incorporation of DLCA-processed images improved diagnostic accuracy for overall (AUC: 0.874/0.845 → 0.958/0.938), central (0.939/0.895 → 0.987/0.972), and peripheral (0.824/0.807 → 0.935/0.912) PE detection (all *p* ≤ 0.003). In suboptimal CTPA, a pulmonary artery attenuation threshold of 130 HU was identified, above which DLCA processing significantly improved PE detection accuracy compared with original images in both readers (*p* < 0.001). **Conclusions**: DLCA processing in suboptimal CTPA significantly enhances image quality and diagnostic accuracy for PE detection, providing a promising strategy to optimize scans without additional contrast or radiation.

## 1. Introduction

CT pulmonary angiography (CTPA) is the gold standard for diagnosing pulmonary embolism (PE). However, suboptimal image quality remains a major challenge, as it may lead to diagnostic inaccuracies and potentially serious consequences in patients with suspected PE [1,2]. Among the factors contributing to suboptimal CTPA quality, poor vascular opacification is one of the most common causes [3,4].

Poor opacification may result from inappropriate bolus timing, transient interruption of contrast inflow due to deep inspiration and breath-holding, hemodynamic alterations, or large patient body habitus [5,6]. Adequate control of contrast administration, proper respiratory instruction, and the use of low kVp settings can help maintain sufficient vascular enhancement in CTPA [6,7]. Nonetheless, a practical solution is needed for cases where CTPA images have already been acquired with inadequate pulmonary artery opacification, as repeating the examination increases the patient’s exposure to contrast media and radiation.

Dual-energy CT (DECT) has shown promise in improving the diagnostic quality of suboptimally enhanced CTPA studies. Low-keV VMI reconstructions, particularly at 40 keV, significantly increase pulmonary artery attenuation, signal-to-noise ratio (SNR), and contrast-to-noise ratio (CNR) compared to standard images [8,9,10]. These enhancements can substantially improve the diagnostic accuracy of PE detection in cases with inadequate contrast opacification [8]. However, not all CTPA studies are performed using DECT technology.

Recently, deep learning has emerged as a powerful tool for improving image quality and, consequently, diagnostic accuracy in CT imaging [11,12,13,14]. Among these approaches, iodine contrast–augmentation algorithms have been developed to enable CT imaging with reduced doses of iodine contrast [15]. Previous studies have applied this technique to CT scans performed under predefined conditions of reduced radiation and iodine load, demonstrating significant improvements in image quality compared with scans without contrast augmentation [16,17,18]. We hypothesized that this iodine contrast–augmentation algorithm could also improve the image quality of CTPA scans that are unintentionally suboptimally enhanced, a clinically relevant challenge in daily practice.

Therefore, this study aimed to evaluate the effect of a deep learning–based iodine contrast–augmentation (DLCA) algorithm on image quality and diagnostic performance for PE detection in suboptimally enhanced CTPA.

## 2. Materials and Methods

**Figure 1:**
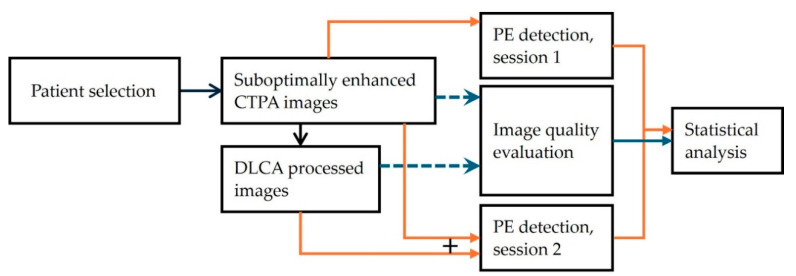
Flow diagram depicting the overall study methodology.

This study was approved by the Institutional Review Board (Gyeongsang National University Changwon Hospital, IRB No. 2025-07-018). The requirement for informed consent was waived due to the retrospective nature of the study and the use of anonymized data. A schematic flow diagram of the overall study methodology is presented in Figure 1.

### 2.1. Study Population

**Figure 2:**
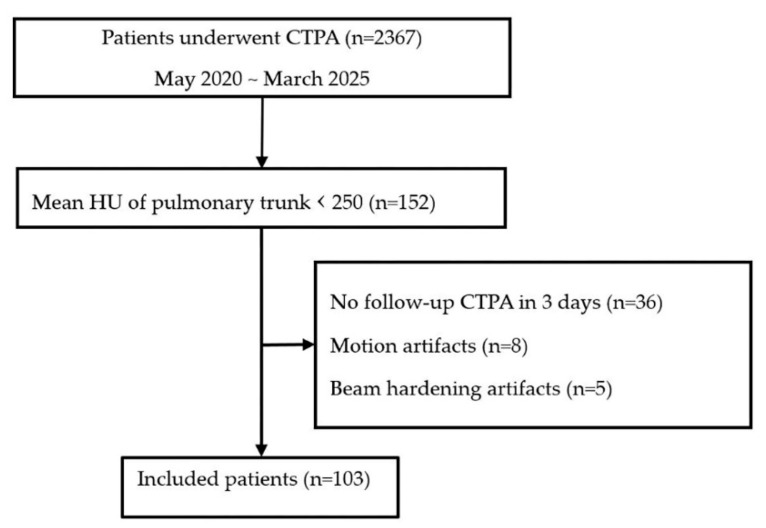
Flowchart illustrating patient selection.

Among 2367 CTPA examinations performed between May 2020 and March 2025, a total of 103 cases with suboptimal pulmonary artery enhancement and available follow-up CTPA within 3 days were retrospectively included (Figure 2). Suboptimal enhancement was defined as a CT attenuation value of less than 250 Hounsfield units (HU) in the pulmonary trunk, based on the image quality assessment reported during CT interpretation. This 250-HU threshold was chosen because it has been considered optimal for confident evaluation of acute PE in previous studies [19,20]. The study cohort comprised 76 male and 27 female patients, with a mean age of 57.6 years (range, 19–92 years).

### 2.2. CT Image Acquisition and Postprocessing

CTPA examinations were performed using one of three CT scanners: IQon (Philips Healthcare, Best, the Netherlands), Aquilion ONE (Canon Medical Systems, Otawara, Japan), or SOMATOM Force (Siemens Healthcare, Forchheim, Germany). CT scanning parameters were as follows: 120 kVp, 140–250 mA, 2 mm slice thickness, 2 mm reconstruction interval, and a smooth reconstruction filter. Images were obtained during a single breath-hold, covering the lung apex to the costophrenic angles in the craniocaudal direction. For contrast enhancement, 70–90 mL of iodinated contrast medium (iohexol; Omnipaque 300, GE Healthcare, Shanghai, China) was injected through an antecubital vein at 3.5–4 mL/s, followed by a 30 mL contrast–saline mixture and a 20 mL saline flush, both at the same rate. Bolus tracking in the pulmonary trunk was used for individualized timing with a threshold of 150 Hounsfield units (HU).

All suboptimal CTPA images were post-processed using a DLCA algorithm (ClariACE, ClariPi Inc., Seoul, Korea) to enhance pulmonary vascular opacification. The DLCA is a commercially available contrast-boosting model developed for low–contrast-dose CT, employing a two-stage U-net architecture. Further technical details of the model have been described in previous studies [16,17].

### 2.3. Image Quality Evaluation

A radiologist with three years of experience measured attenuation values in HU and standard deviations in the pulmonary trunk, back muscle, and room air on the original CTPA images using circular regions of interest (ROIs) placed on axial images. The ROIs were then copied and pasted at the same anatomical locations on the DLCA-processed images. Each measurement was performed three times, and the mean value was recorded as the representative measurement.

The CNR of the pulmonary artery was calculated according to the following equation: CNR = (SI_PA_ − SI_muscle_)/BN, where SI_PA_ represents the mean signal intensity of the pulmonary trunk, SI_muscle_ is the mean signal intensity of the back muscle, and BN denotes the background noise, defined as the standard deviation of attenuation in room air. The SNR was calculated as: SNR = SI_PA_/BN.

### 2.4. PE Detection

Two thoracic radiologists with 29 and 30 years of experience, respectively, and one pulmonologist jointly reviewed all suboptimal CTPA images, along with follow-up CTPA studies, 40-keV VMIs when available, and relevant clinical data to establish the reference standard for PE. Readers assessed PE by identifying the presence of an endoluminal filling defect partially or completely occluding pulmonary arteries [21].

For the comparison of PE detection performance, the pulmonary arteries were divided into 20 anatomical segments (1 trunk, 2 main, 2 interlobar, 5 lobar, 5 segmental, and 5 subsegmental) according to vascular anatomy [22]. PEs from the trunk through the lobar arteries were classified as central, whereas PEs in the segmental and subsegmental arteries were classified as peripheral. PE detection in each segment was independently assessed by two radiologists with 10 and 5 years of experience, first using suboptimal CTPA alone and then, after a 4-week interval, using both the original and DLCA-processed images.

### 2.5. Statistical Analysis

Continuous variables were expressed as means ± standard deviations, and categorical variables as frequencies or percentages. Original and DLCA-processed CT images were compared using paired *t*-tests. Diagnostic accuracy for PE detection was assessed with receiver operating characteristic (ROC) analysis, and pulmonary artery attenuation thresholds were evaluated by comparing area under the curve (AUC) values in 10-HU increments. Inter-reader agreement was assessed using kappa statistics, interpreted as follows: ≤0.20, poor; 0.21–0.40, fair; 0.41–0.60, moderate; 0.61–0.80, substantial; and ≥0.81, almost perfect. Statistical analyses were conducted using SPSS (version 27) and MedCalc (version 23.1.7), with *p* < 0.05 considered statistically significant.

## 3. Results

Among 103 cases with suboptimal enhancement, the causes included incorrect bolus tracking or delayed scan initiation (*n* = 37), transient interruption of contrast flow (*n* = 29), contrast extravasation (*n* = 11), extremely large body habitus (*n* = 10), low injection flow rates (*n* = 9), markedly reduced cardiac function (*n* = 3), technical errors (*n* = 4).

DLCA processing improved pulmonary artery opacification by 57.7% (159.0 ± 25.8 HU → 250.8 ± 46.3 HU; *p* < 0.001) and reduced noise by 56.7% (12.7 ± 2.3 HU → 5.5 ± 1.0 HU; *p* < 0.001). Consequently, SNR and CNR increased markedly (SNR: 13.2 → 47.5; CNR: 8.7 → 37.2; both *p* < 0.001) (Table 1).

According to the standard reference, PEs were identified in 195 segments (83 central, 112 peripheral) across 35 patients, while the remaining 1865 segments from 68 patients were negative.

**Figure 3:**
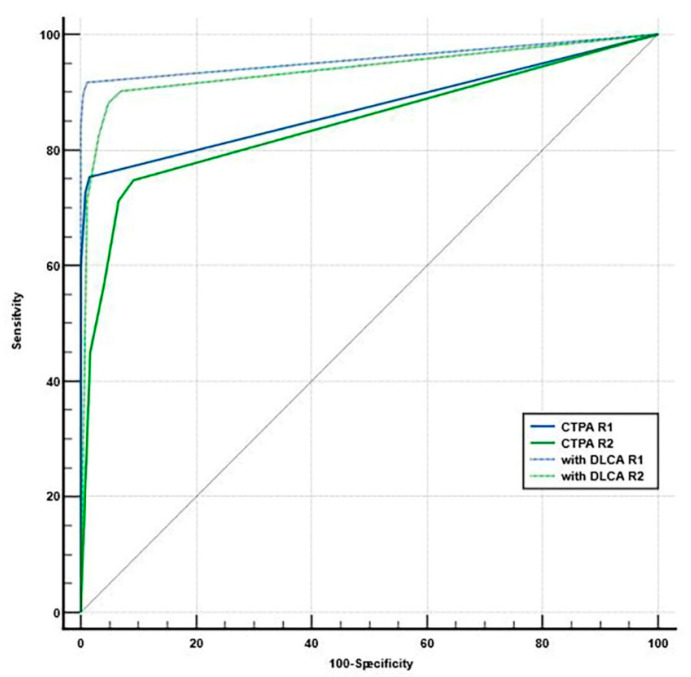
ROC curves demonstrating the diagnostic performance of two readers (R1 and R2) for PE, comparing suboptimal CTPA alone with the addition of DLCA-processed images. The diagonal line represents the no-discrimination threshold.

**Figure 4:**
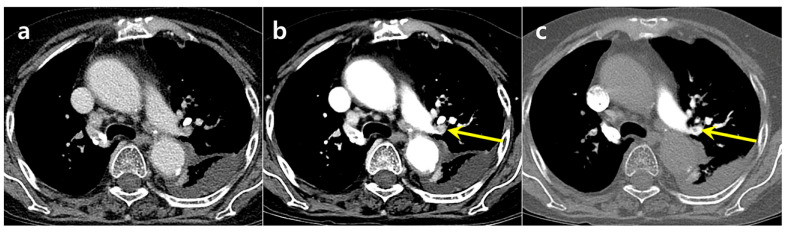
A 93-year-old woman with dyspnea had a PE in the left upper lobe segmental pulmonary artery, which was missed on the suboptimal CTPA (**a**) but detected on the DLCA-processed image (**b**). Follow-up CTPA (**c**) performed the next day confirmed the PE.

**Figure 5:**
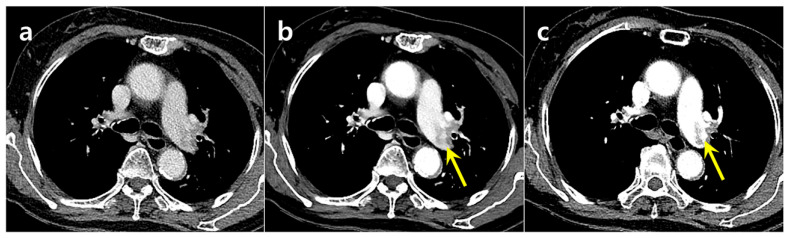
A 72-year-old woman with leg swelling showed a suspicious PE in the left main pulmonary artery on suboptimal CTPA (**a**), which was clearly visualized on the DLCA-processed image (**b**) and confirmed on follow-up CTPA (**c**) the next day.

**Figure 6:**
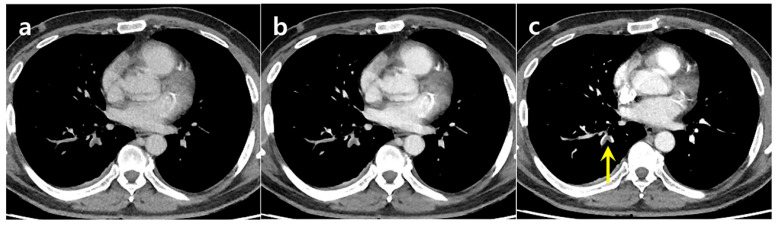
A 49-year-old man with dyspnea had a subsegmental PE in the right lower lobe that was missed on both the suboptimal CTPA (**a**) and the DLCA-processed image (**b**) but detected on follow-up CTPA (**c**) the next day.

For PE detection, the use of DLCA-processed images, in conjunction with the original suboptimal CTPA, significantly improved accuracy for overall, central, and peripheral PE detection (all *p* < 0.05) by both readers (Table 2) (Figure 3, Figure 4, Figure 5 and Figure 6). Inter-reader agreement for PE detection was moderate for both suboptimal CTPA (κ = 0.65) and DLCA-processed images (κ = 0.74).

A pulmonary artery attenuation threshold of 130 HU on the original CTPA was identified, above which DLCA processing enhanced diagnostic performance for PE detection in both readers (*p* < 0.001) (Table 3).

## 4. Discussion

Deep learning-based techniques in thoracic imaging have achieved state-of-the-art performance in image reconstruction and post-processing, enabling advanced imaging capabilities that were previously unattainable [23,24,25,26]. The DLCA algorithm generates contrast-augmented images without requiring specialized hardware such as DECT and was originally developed to reduce the amount of contrast needed in CT imaging. Accordingly, previous studies have applied this algorithm to CT images acquired under predefined low-contrast protocols, including pediatric examinations and adult scans performed with low-kVp settings [15,16,27]. In particular, a CTPA study using DLCA [18] under low-radiation and low-contrast-dose protocols demonstrated that images processed with this algorithm achieved superior vascular opacification, CNR, and SNR compared with CTPA reconstructed using various methods without DLCA processing.

In our study, we specifically evaluated suboptimally enhanced CTPA encountered in routine clinical practice. Consistent with previous reports, DLCA processing improved vascular opacification and reduced image noise, thereby significantly enhancing both CNR and SNR. Moreover, DLCA processing increased diagnostic accuracy for PE detection compared with suboptimal CTPA alone, for overall PEs as well as for both central and peripheral PEs. By contrast, a prior study reported that DLCA was superior for detecting PEs in small vessels, such as segmental and subsegmental arteries [18]. This discrepancy may be explained by their use of low-kVp protocols, in which pulmonary artery attenuation exceeded 250 HU in all groups; consequently, no intergroup differences were observed in detecting more proximal PEs. Our findings therefore extend the applicability of DLCA by demonstrating its value in real-world suboptimal CTPA, a clinically relevant challenge not addressed in previous studies.

In addition, we identified a pulmonary artery attenuation threshold of 130 HU—comparable to that of a venous-phase image—above which DLCA-processed images enhanced diagnostic performance for PE detection, increasing attenuation by approximately 1.6-fold and improving pulmonary artery CNR by about 4.3-fold. Previously, virtual arterial-phase images generated using low-energy VMI reconstruction from DECT have shown potential as a feasible alternative to true CTPA or aortic angiography [28,29,30]. Considering the potential of DLCA demonstrated by our findings, further studies in larger cohorts are warranted to explore whether routine venous-phase chest CT images could serve as an alternative to CTPA when using DLCA, eliminating the need for rapid contrast injection.

Our study has several limitations. First, it was retrospectively designed and included a relatively small number of cases from a single institution, which may limit the generalizability of the findings. Nevertheless, the data reflect real-world practice over an extended period. Importantly, because DLCA can be applied independently of the type of CT scanner used, the algorithm has the potential to be implemented in other institutions and across different clinical settings. Second, the reference standard for PE detection was not based on optimally enhanced CTPA performed on the same day, but rather on follow-up CTPA performed the following 3 days, 40-keV VMI images when available, and clinical evaluation. Third, because of the limited number of positive PE cases, detection performance was evaluated on a per-segment basis.

## 5. Conclusions

Suboptimal opacification is a common cause of limited or nondiagnostic CTPA, which may lead to misdiagnosis, repeat examinations, unnecessary radiation or contrast exposure, and delays in patient care. In this study, the application of the DLCA algorithm significantly improved image quality and diagnostic performance for PE detection in suboptimally enhanced CTPA. These findings underscore the clinical value of DLCA as a practical and widely applicable solution for optimizing CTPA scans without additional contrast media or radiation exposure. Future studies with larger cohorts are warranted to validate these results and further define the role of DLCA in routine clinical practice.

## Figures and Tables

**Table 1:** Quantitative values of pulmonary artery attenuation (HU), CNR, and SNR before and after DLCA processing in suboptimally enhanced CTPA (*n* = 103).

	CTPA	DLCA-Processed Images	*p*-Value
PA attenuation mean HU (range)	159.0 (91–246.6)	250.8 (121–375.5)	<0.001
Noise	12.7 (5.6–17.9)	5.5 (3.3–10)	<0.001
SNR	13.2 (6.6–37.9)	47.5 (23.0–91.9)	<0.001
CNR	8.7 (3.0–29.9)	37.2 (13.4–78.0)	<0.001

DLCA, deep learning-based iodine contrast augmentation algorithm; SNR, signal-to-noise ratio; CNR, contrast-to-noise ratio.

**Table 2:** Diagnostic accuracy for PE detection in CTPA with and without DLCA-processed images by two independent readers.

**Reader 1**	**CTPA Only**	**With DLCA**	***p*-Value**
All PE	0.874 [0.859, 0.888]	0.958 [0.948, 0.996]	<0.001
Central PE	0.939 [0.978, 0.993]	0.987 [0.978, 0.993]	0.003
Peripheral PE	0.824 [0.799, 0.846]	0.935 [0.919, 0.950]	<0.001
**Reader 2**			
All PE	0.845 [0.812, 0.877]	0.938 [0.916, 0.960]	<0.001
Central PE	0.895 [0.874, 0.913]	0.972 [0.960, 0.981]	<0.001
Peripheral PE	0.807 [0.781, 0.830]	0.912 [0.893, 0.929]	<0.001

DLCA, deep learning-based iodine contrast augmentation algorithm.

**Table 3:** Effect of DLCA processing on PE detection at a pulmonary artery attenuation threshold of 130 HU.

	PA Attenuation	CTPA Only	With DLCA	*p*-Value
Reader 1	≥130 HU	0.878 [0.861, 0.892]	0.962 [0.952, 0.971]	<0.001
	<130 HU	0.659 [0.602, 0.712]	0.663 [0.607, 0.717]	0.06
Reader 2	≥130 HU	0.848 [0.830, 0.864]	0.942 [0.930, 0.953]	<0.001
	<130 HU	0.624 [0.567, 0.679]	0.643 [0.585, 0.697]	0.22

DLCA, deep learning-based iodine contrast augmentation algorithm.

## Data Availability

The datasets used and/or analyzed during the current study are available from the corresponding author on reasonable request.

## Abbreviations

The following abbreviations are used in this manuscript:

CTPA	Computed tomography pulmonary angiography
PE	Pulmonary embolism
DECT	Dual-energy computed tomography
SNR	Signal-to-noise ratio
CNR	Contrast-to-noise ratio
DLCA	Deep learning-based iodine contrast augmentation algorithm
ROC	Receiver operating characteristic
ROI	Region of interest
HU	Hounsfield units
